# Retraction: Novel splicing variant c. 208+2T>C in BBS5 segregates with Bardet-Biedl syndrome in an Iranian family by targeted exome sequencing

**DOI:** 10.1042/BSR-2018-1544_RET

**Published:** 2024-10-07

**Keywords:** Bardet-Biedl syndrome, BBS5 gene, Splicing variant, Targeted exome sequencing

This article “Novel splicing variant c. 208+2T>C in BBS5 segregates with Bardet–Biedl syndrome in an Iranian family by targeted exome sequencing” DOI: 10.1042/BSR20181544 is being retracted from *Bioscience Reports* at the request of the Editor-in-Chief and the Editorial Board. This follows receipt of a notification from a reader, alerting the Editorial Board to queries regarding the study's ethical approval. Additionally, concerns were raised regarding duplicated images within this paper, as well as with a previously published paper that shares authors. Specifically:

- Figure 2C OS panel which seems to appear as part of Figure 2B OS panel from Imani et al, 2017 (DOI: 10.1111/jcmm.13454)
- Figure 2E OD panel which appears as Figure 2F OS panel in this paper
- Figure 2F OD panel which appears as Figure 3B from Imani et al, 2017 (DOI: 10.1111/jcmm.13454).

The authors were contacted regarding the concerns and provided relevant documentation relating to the ethical approval of their study which addressed the Journal's queries. The authors state that the incorrect images were accidentally used for Figures 2C OS panel, 2E OD panel, and 2F OD and OS panels, and provided replacement images for these figures for which no further issues were identified. However, given the extent of the duplications identified in the original figures, the Editorial Board stands by the decision to retract. Junjiang Fu agrees with the retraction but stands by the conclusions of the paper. Mohammad Hossein Khosravi and Saber Imani disagree with the retraction. No response was received from the remaining authors.

